# Complete Genome Sequence of *Spiroplasma* sp. TU-14

**DOI:** 10.1128/genomeA.01465-16

**Published:** 2017-01-12

**Authors:** Wen-Sui Lo, Mindia Haryono, Gail E. Gasparich, Chih-Horng Kuo

**Affiliations:** a Institute of Plant and Microbial Biology, Academia Sinica, Taipei, Taiwan; b Molecular and Biological Agricultural Sciences Program, Taiwan International Graduate Program, National Chung Hsing University and Academia Sinica, Taipei, Taiwan; c Graduate Institute of Biotechnology, National Chung Hsing University, Taichung, Taiwan; d Department of Biological Sciences, Towson University, Towson, Maryland, USA; e College of Arts and Sciences, Salem State University, Salem, Massachusetts, USA; f Biotechnology Center, National Chung Hsing University, Taichung, Taiwan

## Abstract

*Spiroplasma* sp. TU-14 was isolated from a contaminated sample of *Entomoplasma lucivorax* PIPN-2^T^ obtained from the International Organization for Mycoplasmology collection. Here, we report the complete genome sequence of this bacterium to facilitate the investigation of its biology and the comparative genomics among *Spiroplasma* spp.

## GENOME ANNOUNCEMENT

The genus *Spiroplasma* contains a group of host-associated bacteria mostly affiliated with various arthropods (1). The strain *Spiroplasma* sp. TU-14 was isolated by Gail Gasparich at Towson University in 2014 from a contaminated sample of *Entomoplasma lucivorax* PIPN-2^T^ (from the International Organization for Mycoplasmology collection; lyophilized on 25 April 1996 after 21 passes from the initial culture). The source of contamination and the ecology of this *Spiroplasma* strain are unknown. Nonetheless, this strain exhibits several intriguing genomic characteristics compared to its relatives. Based on the 16S rDNA sequence, this strain is most closely related to *Spiroplasma insolitum* M55^T^. However, unlike *S. insolitum* and other lineages belonging to the Citri clade, which all contain a large number of plectroviral sequences in their genomes (2–6), our initial survey revealed that *Spiroplasma* sp. TU-14 lacks a prophage sequence and has a relatively small chromosome. To facilitate future investigation into the biology of this bacterium, as well as to improve the taxon sampling of available *Spiroplasma* sequences for comparative genomics and evolutionary studies (7), we determined its complete genome sequence.

The procedures for sequencing, assembly, and annotation were based on those described in our previous studies on *Spiroplasma* genomes (4–6, 8–15). Briefly, we used the Illumina MiSeq platform to generate 301-bp reads from one paired-end library (~550-bp insert, ~322 Mb, ~269-fold coverage). The initial *de novo* assembly was performed using Velvet version 1.2.10 (16). Subsequently, PAGIT version 1 (17) was used to assist an iterative process for improving the assembly. For each iteration, the raw reads were mapped to the assembly using BWA version 0.7.12 (18), programmatically checked using the MPILEUP program in SAMTOOLS package version 1.2 (19), and visually inspected using IGV version 2.3.57 (20). Polymorphic sites and gaps were corrected based on the mapped reads. The process was repeated until the complete genome sequence was obtained. The programs RNAmmer (21), tRNAscan-SE (22), and Prodigal (23) were used for gene prediction. The gene names and product descriptions were first annotated based on the homologous genes in other *Spiroplasma* genomes (4–6, 8–15) as identified by OrthoMCL (24). Subsequent manual curation was based on BLASTp (25) searches against the NCBI nonredundant database (26) and the KEGG database (27, 28). Putative clustered regularly interspaced short palindromic repeats (CRISPRs) were identified using CRISPRFinder (29).

The circular chromosome of *Spiroplasma* sp. TU-14 is 1,199,640 bp in size and has a G+C content of 28.7%, no plasmid was found. The first version of the annotation includes one set of 16S-23S-5S rRNA genes, 32 tRNA genes (covering all 20 amino acids), 1,036 protein-coding genes, and four pseudogenes. No putative plectroviral sequence or CRISPR element was found.

### Accession number(s).

The complete genome sequence of *Spiroplasma* sp. TU-14 has been deposited at DDBJ/EMBL/GenBank under the accession number CP017658.

## References

[1] Gasparich GE, Whitcomb RF, Dodge D, French FE, Glass J, Williamson DL 2004 The genus *Spiroplasma* and its non-helical descendants: phylogenetic classification, correlation with phenotype and roots of the *Mycoplasma mycoides* clade. Int J Syst Evol Microbiol 54:893–918. doi:10.1099/ijs.0.02688-0. 15143041

[2] Carle P, Saillard C, Carrère N, Carrère S, Duret S, Eveillard S, Gaurivaud P, Gourgues G, Gouzy J, Salar P, Verdin E, Breton M, Blanchard A, Laigret F, Bové JM, Renaudin J, Foissac X 2010 Partial chromosome sequence of *Spiroplasma citri* reveals extensive viral invasion and important gene decay. Appl Environ Microbiol 76:3420–3426. doi:10.1128/AEM.02954-09. 20363791 PMC2876439

[3] Alexeev D, Kostrjukova E, Aliper A, Popenko A, Bazaleev N, Tyakht A, Selezneva O, Akopian T, Prichodko E, Kondratov I, Chukin M, Demina I, Galyamina M, Kamashev D, Vanyushkina A, Ladygina V, Levitskii S, Lazarev V, Govorun V 2012 Application of *Spiroplasma* *melliferum* proteogenomic profiling for the discovery of virulence factors and pathogenicity mechanisms in host-associated spiroplasmas. J Proteome Res 11:224–236. doi:10.1021/pr2008626. 22129229

[4] Lo WS, Chen LL, Chung WC, Gasparich GE, Kuo CH 2013 Comparative genome analysis of *Spiroplasma* *melliferum* IPMB4A, a honeybee-associated bacterium. BMC Genomics 14:22. doi:10.1186/1471-2164-14-22. 23324436 PMC3563533

[5] Ku C, Lo WS, Chen LL, Kuo CH 2013 Complete genomes of two dipteran-associated spiroplasmas provided insights into the origin, dynamics, and impacts of viral invasion in *Spiroplasma*. Genome Biol Evol 5:1151–1164. doi:10.1093/gbe/evt084. 23711669 PMC3698928

[6] Paredes JC, Herren JK, Schüpfer F, Marin R, Claverol S, Kuo CH, Lemaitre B, Béven L 2015 Genome sequence of the *Drosophila melanogaster* male-killing *Spiroplasma* strain MSRO endosymbiont. mBio 6:e02437-14. doi:10.1128/mBio.02437-14. 25827421 PMC4453565

[7] Lo WS, Huang YY, Kuo CH 2016 Winding paths to simplicity: genome evolution in facultative insect symbionts. FEMS Microbiol Rev 40:855–874. doi:10.1093/femsre/fuw028. PMC5091035 28204477

[8] Lo WS, Ku C, Chen LL, Chang TH, Kuo CH 2013 Comparison of metabolic capacities and inference of gene content evolution in mosquito-associated *Spiroplasma* *diminutum* and *S.* *taiwanense*. Genome Biol Evol 5:1512–1523. doi:10.1093/gbe/evt108. 23873917 PMC3762197

[9] Ku C, Lo WS, Chen LL, Kuo CH 2014 Complete genome sequence of *Spiroplasma apis* B31^T^ (ATCC 33834), a bacterium associated with May disease of honeybees (*Apis mellifera*). Genome Announc 2(1):e01151-13. doi:10.1128/genomeA.01151-13. 24407648 PMC3886961

[10] Chang TH, Lo WS, Ku C, Chen LL, Kuo CH 2014 Molecular evolution of the substrate utilization strategies and putative virulence factors in mosquito-associated *Spiroplasma* species. Genome Biol Evol 6:500–509. doi:10.1093/gbe/evu033. 24534435 PMC3971584

[11] Lo WS, Gasparich GE, Kuo CH 2015 Found and lost: the fates of horizontally acquired genes in arthropod-symbiotic *Spiroplasma*. Genome Biol Evol 7:2458–2472. doi:10.1093/gbe/evv160. 26254485 PMC4607517

[12] Lo WS, Lai YC, Lien YW, Wang TH, Kuo CH 2015 Complete genome sequence of *Spiroplasma* *litorale* TN-1^T^ (DSM 21781), a bacterium isolated from a green-eyed horsefly (*Tabanus nigrovittatus*). Genome Announc 3(5):e01116-15. doi:10.1128/genomeA.01116-15. 26430038 PMC4591310

[13] Lo WS, Liu PY, Kuo CH 2015 Complete genome sequence of *Spiroplasma* *cantharicola* CC-1^T^ (DSM 21588), a bacterium isolated from soldier beetle (*Cantharis carolinus*). Genome Announc 3(5):e01253-15. doi:10.1128/genomeA.01253-15. 26494686 PMC4616193

[14] Lo WS, Gasparich GE, Kuo CH 2016 Complete genome sequence of *Spiroplasma* *turonicum* Tab4c^T^, a bacterium isolated from horse flies (*Haematopota* sp.). Genome Announc 4(5):e01010-16. doi:10.1128/genomeA.01010-16. 27660788 PMC5034139

[15] Shen WY, Lo WS, Lai YC, Kuo CH 2016 Complete genome sequence of *Spiroplasma* *helicoides* TABS-2^T^ (DSM 22551), a bacterium isolated from a horsefly (*Tabanus abactor*). Genome Announc 4(5):e01201-16. doi:10.1128/genomeA.01201-16. 27795290 PMC5073277

[16] Zerbino DR, Birney E 2008 Velvet: algorithms for *de* *novo* short read assembly using de Bruijn graphs. Genome Res 18:821–829. doi:10.1101/gr.074492.107. 18349386 PMC2336801

[17] Swain MT, Tsai IJ, Assefa SA, Newbold C, Berriman M, Otto TD 2012 A post-assembly genome-improvement toolkit (PAGIT) to obtain annotated genomes from contigs. Nat Protoc 7:1260–1284. doi:10.1038/nprot.2012.068. 22678431 PMC3648784

[18] Li H, Durbin R 2009 Fast and accurate short read alignment with Burrows–Wheeler transform. Bioinformatics 25:1754–1760. doi:10.1093/bioinformatics/btp324. 19451168 PMC2705234

[19] Li H, Handsaker B, Wysoker A, Fennell T, Ruan J, Homer N, Marth G, Abecasis G, Durbin R, 1000 Genome Project Data Processing Subgroup 2009 The sequence alignment/map format and SAMtools. Bioinformatics 25:2078–2079. doi:10.1093/bioinformatics/btp352. 19505943 PMC2723002

[20] Robinson JT, Thorvaldsdóttir H, Winckler W, Guttman M, Lander ES, Getz G, Mesirov JP 2011 Integrative genomics viewer. Nat Biotechnol 29:24–26. doi:10.1038/nbt.1754. 21221095 PMC3346182

[21] Lagesen K, Hallin P, Rødland EA, Staerfeldt H-H, Rognes T, Ussery DW 2007 RNAmmer: consistent and rapid annotation of ribosomal RNA genes. Nucleic Acids Res 35:3100–3108. doi:10.1093/nar/gkm160. 17452365 PMC1888812

[22] Lowe TM, Eddy SR 1997 TRNAscan-SE: a program for improved detection of transfer RNA genes in genomic sequence. Nucleic Acids Res 25:955–964. doi:10.1093/nar/25.5.955. 9023104 PMC146525

[23] Hyatt D, Chen GL, LoCascio PF, Land ML, Larimer FW, Hauser LJ 2010 Prodigal: prokaryotic gene recognition and translation initiation site identification. BMC Bioinformatics 11:119. doi:10.1186/1471-2105-11-119. 20211023 PMC2848648

[24] Li L, Stoeckert CJ, Roos DS 2003 OrthoMCL: identification of ortholog groups for eukaryotic genomes. Genome Res 13:2178–2189. doi:10.1101/gr.1224503. 12952885 PMC403725

[25] Camacho C, Coulouris G, Avagyan V, Ma N, Papadopoulos J, Bealer K, Madden TL 2009 BLAST+: architecture and applications. BMC Bioinformatics 10:421. doi:10.1186/1471-2105-10-421. 20003500 PMC2803857

[26] Clark K, Karsch-Mizrachi I, Lipman DJ, Ostell J, Sayers EW 2016 GenBank. Nucleic Acids Res 44:D67–D72. doi:10.1093/nar/gkv1276. 26590407 PMC4702903

[27] Kanehisa M, Goto S 2000 KEGG: Kyoto Encyclopedia of Genes and Genomes. Nucleic Acids Res 28:27–30. doi:10.1093/nar/28.1.27. 10592173 PMC102409

[28] Kanehisa M, Goto S, Furumichi M, Tanabe M, Hirakawa M 2010 KEGG for representation and analysis of molecular networks involving diseases and drugs. Nucleic Acids Res 38:D355–D360. doi:10.1093/nar/gkp896. 19880382 PMC2808910

[29] Grissa I, Vergnaud G, Pourcel C 2007 CRISPRFinder: a web tool to identify clustered regularly interspaced short palindromic repeats. Nucleic Acids Res 35:W52–W57. doi:10.1093/nar/gkm360. 17537822 PMC1933234

